# Mutation of the co-chaperone Tsc1 in bladder cancer diminishes Hsp90 acetylation and reduces drug sensitivity and selectivity

**DOI:** 10.18632/oncotarget.27217

**Published:** 2019-10-08

**Authors:** Mark R. Woodford, Michael Hughes, Rebecca A. Sager, Sarah J. Backe, Alexander J. Baker-Williams, Michael S. Bratslavsky, Joseph M. Jacob, Oleg Shapiro, Michael Wong, Gennady Bratslavsky, Dimitra Bourboulia, Mehdi Mollapour

**Affiliations:** ^1^ Department of Urology, SUNY Upstate Medical University Syracuse, NY 13210, USA; ^2^ Department of Biochemistry and Molecular Biology, SUNY Upstate Medical University Syracuse, NY 13210, USA; ^3^ Upstate Cancer Center, SUNY Upstate Medical University Syracuse, NY 13210, USA; ^4^ College of Medicine, SUNY Upstate Medical University Syracuse, NY 13210, USA; ^5^ Department of Neurology, Washington University School of Medicine, Saint Louis, MO 63110, USA; ^6^ Hope Center for Neurological Disorders, Washington University School of Medicine, Saint Louis, MO 63110, USA

**Keywords:** tuberous sclerosis complex (TSC), Tsc1 (Hamartin), Tsc2 (Tuberin), heat shock protein (Hsp90), bladder cancer

## Abstract

The molecular chaperone Heat shock protein 90 (Hsp90) is essential for the folding, stability, and activity of several drivers of oncogenesis. Hsp90 inhibitors are currently under clinical evaluation for cancer treatment, however their efficacy is limited by lack of biomarkers to optimize patient selection. We have recently identified the tumor suppressor tuberous sclerosis complex 1 (Tsc1) as a new co-chaperone of Hsp90 that affects Hsp90 binding to its inhibitors. Highly variable mutations of TSC1 have been previously identified in bladder cancer and correlate with sensitivity to the Hsp90 inhibitors. Here we showed loss of TSC1 leads to hypoacetylation of Hsp90-K407/K419 and subsequent decreased binding to the Hsp90 inhibitor ganetespib. Pharmacologic inhibition of histone deacetylases (HDACs) restores acetylation of Hsp90 and sensitizes Tsc1-mutant bladder cancer cells to ganetespib, resulting in apoptosis. Our findings suggest that TSC1 status may predict response to Hsp90 inhibitors in patients with bladder cancer, and co-targeting HDACs can sensitize tumors with Tsc1 mutations to Hsp90 inhibitors.

## INTRODUCTION

Over 80,000 people will be diagnosed with bladder cancer and approximately 18,000 patients will die from this disease in the United States in 2019 [1]. The vast majority of bladder cancer cases are urothelial cell carcinomas (90%) followed by squamous cell carcinomas (5%) with the remaining cases being made up of rare entities including sarcoma and small cell carcinoma [2]. Bladder cancer has the highest lifetime cost of all cancers, which is attributed to high recurrence rates and the invasive monitoring required in the management of this disease [3]. Depending on stage, standard of care in management of bladder cancer may include local resection, chemotherapy, immunotherapy, radiotherapy, or partial or radical cystectomy [4]. Advancements in the treatment of metastatic disease have been few, with alternatives to chemotherapeutic mainstay therapies offering limited survival benefit [5, 6].

The molecular chaperone heat shock protein-90 (Hsp90) is essential for the stabilization and activation of many oncogenic proteins, known as clients, involved in malignant transformation of tumor cells [7–9]. Hsp90 chaperone function is coupled to its ability to bind and hydrolyze ATP [10–12]. This ATPase activity provides directionality to the Hsp90 chaperone cycle, which is tightly regulated by both co-chaperone proteins and post-translational modifications [13–15]. Numerous small molecules have been identified that compete with ATP for binding to the amino-domain of Hsp90 and inhibit its chaperone function, leading to the degradation of many client proteins involved in tumorigenesis [7]. There are currently 7 Hsp90 inhibitors in clinical trials (https://www.clinicaltrials.gov/), and there is an urgent need to identify biomarkers to help identify those patients that would respond favorably to treatment with Hsp90 inhibitors [16].

We have recently identified the tumor suppressor *TSC1* as a novel regulator/co-chaperone of Hsp90 important for the folding and stability of numerous kinase and non-kinase clients including Tsc2 protein (tuberin) [17]. Tsc2 protein has a GTPase-activating function and in complex with Tsc1 protein (hamartin) and possibly Hsp90 acts as a negative regulator of AMPK/mTOR signaling [18–20]. Additionally, Tsc1 assists in the deceleration of Hsp90 ATPase activity and the Hsp90 chaperone cycle, and Tsc1 expression increases Hsp90 binding to its inhibitors [17].

Mutation and inactivation of the tumor suppressor *TSC1* has been found in approximately 15% of bladder cancers and loss of heterozygosity of a region spanning the *TSC1* locus at 9q34 has been seen in roughly 54% of bladder cancers [21–26]. We therefore hypothesized that mutation and inactivation of *TSC1* in bladder cancer cells leads to decreased sensitivity to Hsp90 inhibitors. Our data supported this hypothesis, and we mechanistically demonstrated that mutation and loss of *TSC1* in bladder cancer cells causes hypoacetylation of Hsp90-K407/K419 and subsequent decreased binding of Hsp90 to its inhibitor ganetespib. Pharmacologic inhibition of histone deacetylases (HDACs) restores acetylation of Hsp90 and sensitizes Tsc1-mutant bladder cancer cells to ganetespib, resulting in apoptosis. Our results suggest that Tsc1 status can predict response to Hsp90 inhibition in bladder cancer patients and further provide a strategy to co-target HDACs and Hsp90 in bladder cancers with mutation in *TSC1.*

## RESULTS

### Tsc1 expression determines Hsp90 inhibitor accumulation and sensitivity in bladder cancer cells

**Figure 1:**
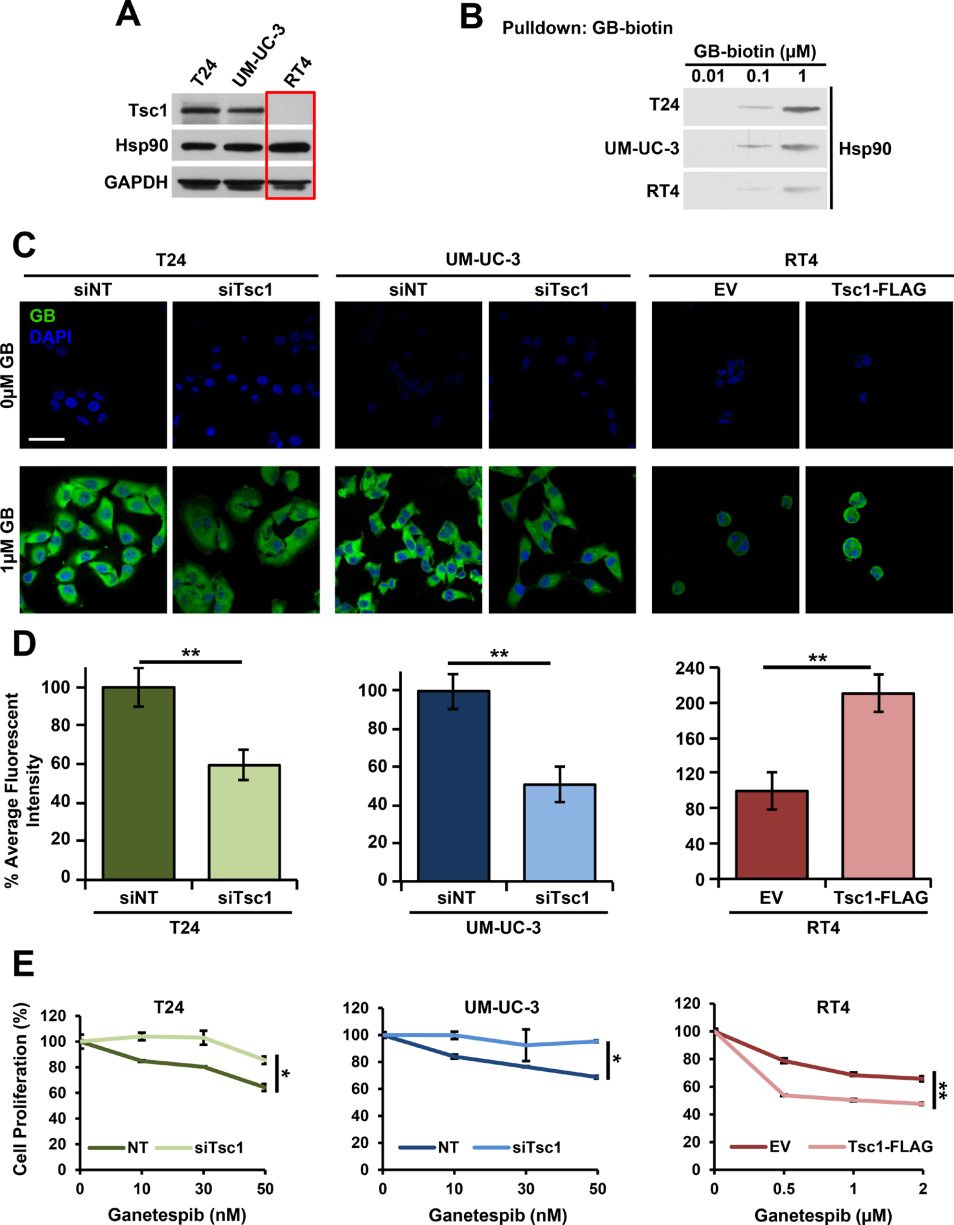
Tsc1 expression determines Hsp90 inhibitor accumulation and sensitivity in bladder cancer cells. (**A**) Tsc1 status in T24, UM-UC-3 and RT4 bladder cancer cell lines was assessed by immunoblot. GAPDH was used as a loading control. (**B**) Lysates from Figure 1A were challenged with biotinylated-ganetespib. Binding of Hsp90 from T24, UM-UC-3 and RT4 cells to biotinylated-ganetespib was examined by immunoblot. (**C**) *TSC1* was targeted by siRNA in T24 and UM-UC-3 cells and Tsc1-FLAG was transiently expressed in RT4 cells. Representative confocal microscopy images of these cells treated for 4hr with BODIPY-ganetespib at the indicated concentrations and stained with DAPI. Scale bar = 50 μm. (**D**) Quantification of average fluorescence intensity of BODIPY-ganetespib in (C). A Student’s *t*-test was performed to assess statistical significance (^**^ *p* < 0.01). (**E**) *TSC1* was targeted by siRNA in T24 (left) and UM-UC-3 (center) and Tsc1-FLAG was transiently expressed in RT4 (right) cells for 48 hr. Following this, cells were treated for an additional 72 hr with the indicated concentrations of ganetespib. Cell proliferation was assessed by WST proliferation assay. A Student’s *t*-test was performed to assess statistical significance (^*^ *p* < 0.05; ^**^ *p* < 0.01).

Our previous study has shown that the presence of Tsc1 co-chaperone, which interacts with Hsp90 through the C-terminus of Tsc1, enhances Hsp90 binding to its inhibitors in cells [17]. Here, we used biotinylated inhibitor ganetespib (GB-biotin) to determine the binding affinity of Hsp90 from T24 and UM-UC-3 cells, which express wild-type (WT) *TSC1* as well as RT4 cells that have a *TSC1* mutation (1669delC), which leads to a frame shift and premature stop codon, rendering the protein product (Tsc1-L557Cfs) unstable (Figure 1A, 1B; Supplementary Figure 1A) [27]. Our data showed that Hsp90 binding was significantly reduced in *TSC1* mutated RT4 cells compared to *TSC1* WT T24 and UM-UC-3 bladder cancer cells (Figure 1B, Supplementary Figure 1B). We have further demonstrated that presence of Tsc1 facilitates accumulation of fluorescently-tagged Hsp90 inhibitor, BODIPY-ganetespib, in bladder cancer cells after 4 hours of treatment (Figure 1C, 1D; Supplementary Figure 1C–1E). This ganetespib accumulation was reduced when *TSC1* was silenced by siRNA in T24 and UM-UC-3 cells (Figure 1C, 1D; Supplementary Figure 1C, 1D). Conversely, re-expression of WT Tsc1 in RT4 cells restored uptake and retention of ganetespib in these bladder cancer cells (Figure 1C, 1D; Supplementary Figure 1C, 1E). In addition to the effect on inhibitor accumulation, *TSC1* expression also significantly sensitized RT4 bladder cancer cells to Hsp90 inhibitor as evidenced by WST proliferation assay (Figure 1E). Conversely, silencing of *TSC1* in T24 and UM-UC-3 cells reversed their sensitivity to ganetespib. Taken together, these data show that presence of Tsc1 enhances bladder cancer cell sensitivity and uptake of Hsp90 inhibitors.

### Tsc1 facilitates acetylation of Hsp90

**Figure 2:**
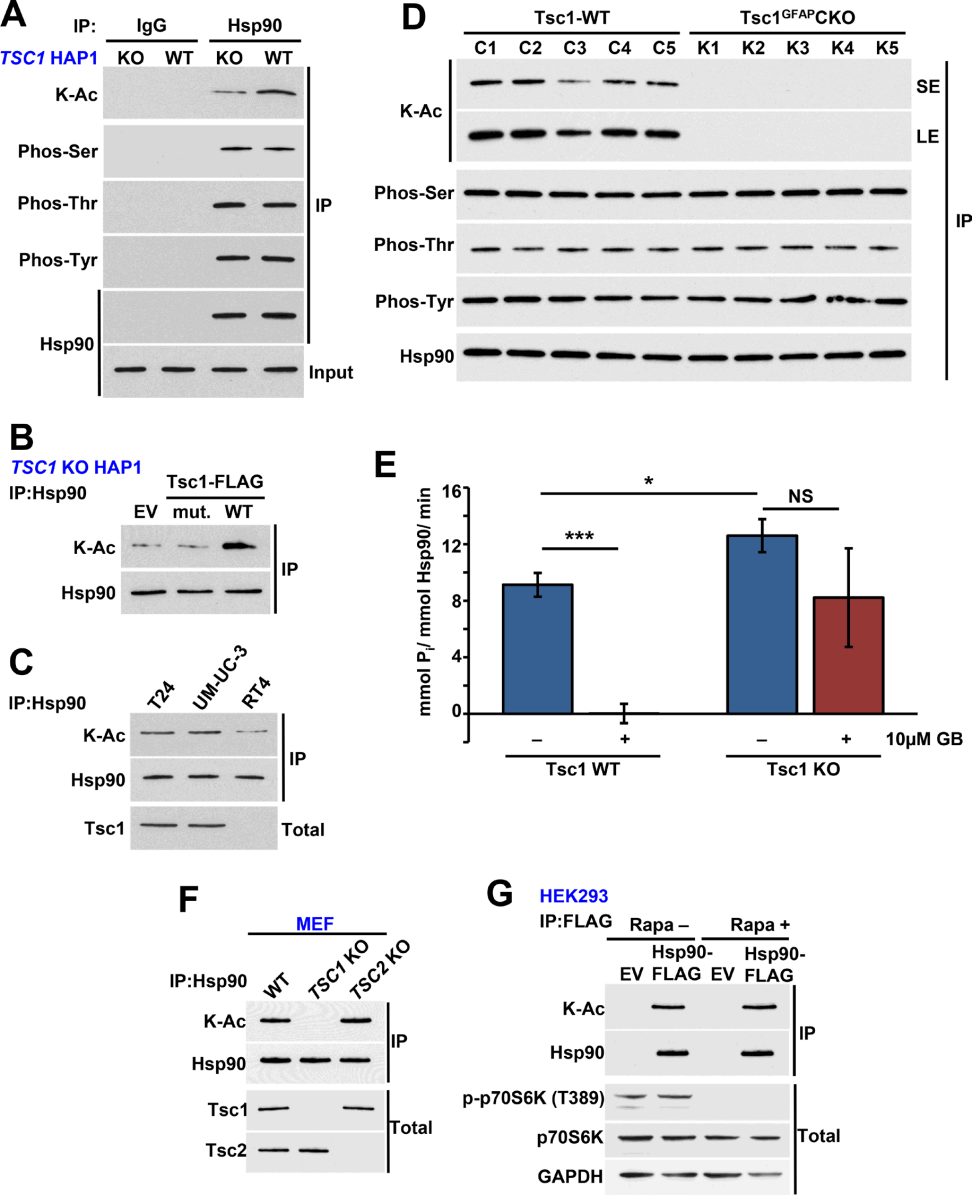
Tsc1 facilitates acetylation of Hsp90. (**A**) Hsp90 was immunoprecipitated from *TSC1* KO and *TSC1* WT HAP1 cells and its serine, threonine, and tyrosine phosphorylation and lysine acetylation were examined by immunoblot. IgG was used as a control. (**B**) *TSC1* KO HAP1 cells were transiently transfected with EV, Tsc1-TW-FLAG or Tsc1-L557Cfs-FLAG (mut.), the mutation found in the RT4 cell line. Acetylation of Hsp90 was then determined by immunoprecipitation and immunoblot. (**C**) Acetylation status of Hsp90 in T24, UM-UC-3 and RT4 determined by immunoprecipitation and immunoblot. (**D**) Hsp90 was immunoprecipitated from WT and astrocyte-specific *TSC1* KO mouse brain lysate and serine, threonine, and tyrosine phosphorylation and lysine acetylation of Hsp90 were assessed by immunoblot. LE=long exposure, SE=short exposure. (**E**) ATPase activity of Hsp90 isolated from *TSC1* WT and *TSC1* KO HAP1 cells. 10 μM ganetespib was used as a control. A Student’s t-test was performed to assess statistical significance (n. s., not significant; ^*^ *p* < 0.05; ^***^ *p* < 0.001). (**F**) Endogenous Hsp90 was isolated from MEF WT, Tsc1 KO or Tsc2 KO cells and acetylation was examined by immunoblot. Tsc1 and Tsc2 status was also examined. (**G**) HEK293 cells transiently expressing Hsp90α-WT-FLAG and empty plasmid (EV) was treated with rapamycin (20 nM) for 1 hr. Hsp90α-WT-FLAG was immunoprecepitated and its acetylation was evaluated by immunoblot. p70S6K and p-p70S6K (T389) were used to examine the inhibition of mTOR pathway.

Previous studies from our lab and others have shown that post-translation modification (PTM) of Hsp90 impacts its binding to as well as sensitizes cells to Hsp90 inhibitors [15, 28–30]. We therefore asked whether absence of Tsc1 impacts the PTM of Hsp90. We showed hypoacetylation of Hsp90 in CRISPR/Cas9 *TSC1* KO HAP1 compared to WT HAP1 cells (Figure 2A; Supplementary Figure 2A). Interestingly, lack of *TSC1* did not affect phosphorylation of Hsp90 on serine, threonine, or tyrosine residues (Figure 2A). Expression of WT *TSC1* in *TSC1* KO HAP1 cells restored acetylation of Hsp90, however we did not obtain similar results upon overexpression of Tsc1-L557Cfs (Figure 2B). We made a similar observation in RT4 cells, which contain the Tsc1-L557Cfs mutation and showed hypoacetylation of Hsp90 relative to WT Tsc1 containing T24 and UM-UC-3 cells (Figure 2C). It is noteworthy that overexpression and stabilization of this mutant does not restore its binding to Hsp90, likely because it lacks the C-terminal region of Tsc1 that binds to Hsp90 (Supplementary Figure 2B) [17].

Our previous work has shown that the conditional knockout of *TSC1* in mouse brain (Tsc1^GFAP^CKO) caused a significant increase in ATPase activity compared to the control samples [17]. Using those samples, we showed that Hsp90 is also hypoacetylated in Tsc1^GFAP^CKO tissue (Figure 2D, LE=long exposure, SE=short exposure). Therefore, we asked whether a similar phenomenon was present in the *TSC1* KO HAP1 cells. In agreement with our previously published data [17], we found that Hsp90 isolated from *TSC1* KO cells displayed increased ATPase activity compared to Hsp90 from WT HAP1 cells (Figure 2E; Supplementary Figure 2C, 2D).

Finally, Tsc1-Tsc2 are involved in negatively regulating the mTOR signaling pathways. We therefore asked whether the effect of the Tsc1 mutation on Hsp90 acetylation is independent of mTOR pathway. Endogenous Hsp90 isolated from *TSC2* knock out mouse embryonic fibroblast cells (MEF) had a similar level acetylation as the Hsp90 isolated WT MEF cells (Figure 2F). As expected Hsp90 from *TSC1* KO MEF cells was hypoacetylated (Figure 2F). Additionally treating HEK293 cells with rapamycin did not impact acetylation of Hsp90 (Figure 2G) suggesting the effect of Tsc1 mutation or loss on Hsp90 acetylation is not mediated through the mTOR pathway.

Taken together, our results show that mutation and loss of Tsc1 stability and expression leads to hypoacetylation of Hsp90 and enhanced Hsp90 ATPase activity.

### Tsc1 facilitates acetylation of Hsp90-K407/K419

**Figure 3:**
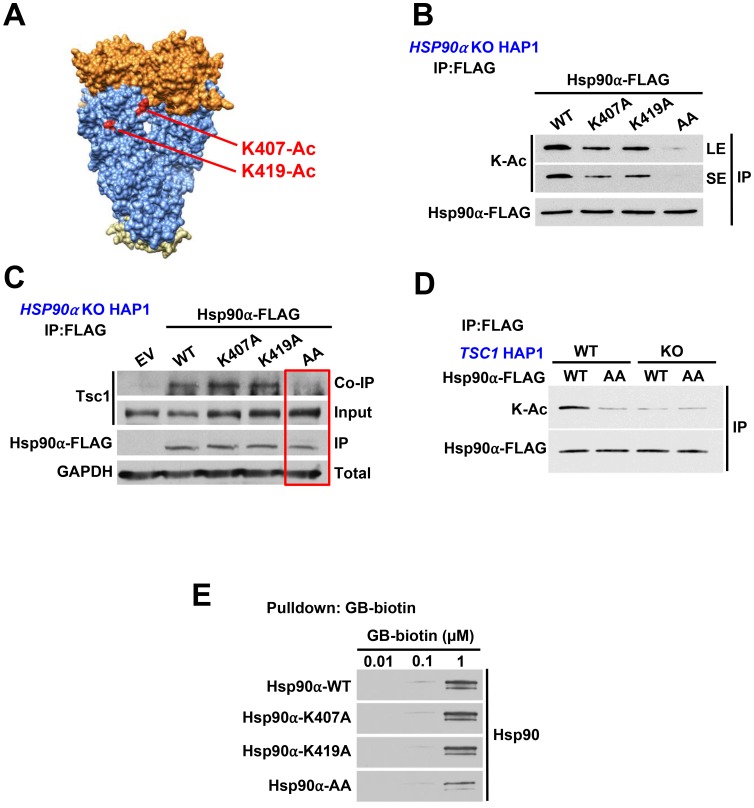
Tsc1 facilitates acetylation of Hsp90-K407/K419. (**A**) Structure of an Hsp90 dimer (PDB:2CG9). K407 and K419 are highlighted on one protomer in red. (**B**) FLAG-tagged Hsp90α-WT or non-acetylatable mutants K407A, K419A, or K407/K419A were transfected into *HSP90α* KO HAP1 cells. Acetylation status was determined by immunoprecipitation and immunoblot. LE=long exposure, SE=short exposure. (**C**) Tsc1 interaction with Hsp90 and acetylation mutants was assessed by FLAG immunoprecipitation of Hsp90 from lysates in (B). (**D**) Hsp90 acetylation was determined by immunoprecipitation and immunoblot of Hsp90α-WT-FLAG and Hsp90α-K407/K419A-FLAG from *TSC1* WT and *TSC1* KO HAP1 cells. (**E**) Lysates from Figure 3B were challenged with biotinylated-ganetespib. Binding of Hsp90α-FLAG from these lysates to biotinylated-ganetespib was examined by immunoblot.

Hypoacetylation of Hsp90 leads to its elevated ATPase activity; we therefore reasoned that lysine residues within the catalytic region of Hsp90 are candidates for PTMs. We identified two lysine sites (K407/K419) within this region that have been reported to be subject to acetylation (https://www.phosphosite.org//homeAction.action; Figure 3A). We mutated these lysine residues to non-acetylatable alanine in Hsp90α individually and in combination and exogenously expressed them in CRISPR/Cas9 *HSP90AA1* KO HAP1 cells (Supplementary Figure 3A). Our data showed a reduction in acetylation of Hsp90α-FLAG-K407A and K419A and significant hypoacetylation of Hsp90α-FLAG-K407A/K419A suggesting that both of these residues are subject to acetylation (Figure 3B). It is noteworthy that K407 and K419 are not the only lysine sites that are acetylated on Hsp90α. Longer-exposure of immunoblots in Figure 3B confirms acetylation of Hsp90α and its mutants. Using anti-FLAG M2 agarose, we immunoprecipitated WT Hsp90α-FLAG as well as the lysine to alanine mutants from these cells and showed that Tsc1 interaction was completely abrogated in the K407A/K419A (AA) mutant (Figure 3C). We further demonstrated that Hsp90 was hypoacetylated in CRISPR/Cas9 *TSC1* KO HAP1 cells to the same levels as the K407A/K419A (AA) mutant (Figure 3D), suggesting these lysine residues were the only sites affected as the result of *TSC1* deletion. Finally, we demonstrated a significant reduction in binding of Hsp90α-K407A/K419A to biotinylated ganetespib, which demonstrates that acetylation of these two residues are important for Hsp90 inhibitor binding (Figure 3E, Supplementary Figure 3B). In aggregate, our results demonstrate that lack of Tsc1 expression leads to hypoacetylation of Hsp90 on K407/K419 and subsequent reduced binding of Hsp90 to its inhibitors.

### HDAC inhibition rescues Hsp90 acetylation in TSC1-knock out cells

**Figure 4:**
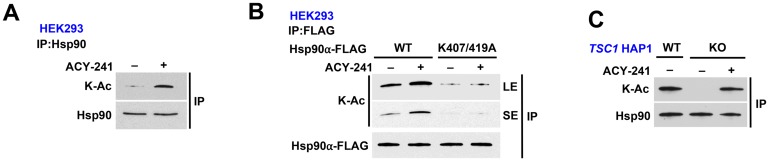
HDAC inhibition rescues Hsp90 acetylation in *TSC1*-knock out cells. (**A**) Endogenous Hsp90 was immunoprecipitated from HEK293 cells treated with or without 1 μM pan-HDAC inhibitor ACY-241 for 16 hr and its acetylation status was determined by immunoblot. (**B**) Hsp90α-WT-FLAG and non-acetylatable mutant K407/K419A were transiently expressed in HEK293 cells in the presence or absence of 1 μM ACY-241 for 16 hr. Acetylation status was evaluated by immunoprecipitation followed by immunoblot. LE=long exposure, SE=short exposure. (**C**) *TSC1* WT HAP1 cells and *TSC1* KO HAP1 cells were treated with or without 1μM ACY-241 for 16 hr. Endogenous Hsp90 was isolated and its acetylation status was examined by immunoblot.

Histone deacetylases (HDAC) modify Hsp90 [14, 31, 32], therefore we treated HEK293 cells with an HDAC inhibitor ACY-241 (acts as a pan-HDAC inhibitor at high dosage) for 16 hours, and showed hyperacetylation of Hsp90 (Figure 4A; Supplementary Figure 4A). We further utilized this pan-HDAC inhibitor to demonstrate that treatment of HEK293 cells expressing Hsp90α-K407A/K419A does not increase the acetylation on this mutant (Figure 4B). This suggests that the effect of HDAC inhibition on Hsp90 is likely only through K407 and K419. Finally, endogenous Hsp90 from *TSC1* KO HAP1 cells is hypoacetylated but treating these cells with the HDAC inhibitor ACY-241 rescued Hsp90 acetylation (Figure 4C, Supplementary Figure 4B).

### HDAC inhibition synergizes with Hsp90 inhibition to induce apoptosis in bladder cancer

**Figure 5:**
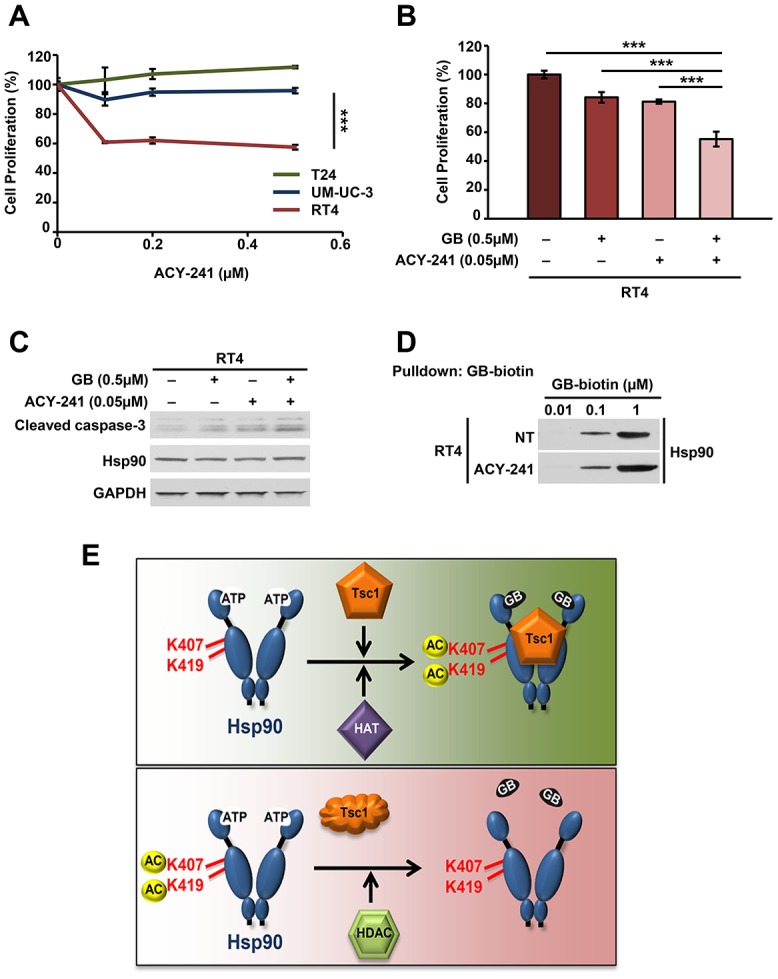
HDAC inhibition synergizes with Hsp90 inhibition to induce apoptosis in bladder cancer. (**A**) Sensitivity of T24, UM-UC-3 and RT4 cells to increasing concentrations of ACY-241 was determined by WST proliferation assay. A Student’s t-test was performed to assess statistical significance (^***^ *p* < 0.001). (**B**) RT4 cells were treated with or without 0.5μM ACY-241 for 16 hr followed by 0.5μM ganetespib for an additional 48 hr. Cell viability was then determined by WST proliferation assay. A Student’s t-test was performed to assess statistical significance (^***^ *p* < 0.001). (**C**) Immunoblot of apoptotic marker cleaved caspase-3 in RT4 cells from (B). (**D**) Hsp90 binding to biotinylated-ganetespib was examined in RT4 cells treated with or without 1 μM ACY-241 for 16 hr. (**E**) Impact of the co-chaperone Tsc1 on Hsp90 binding to its inhibitors. Tsc1 facilitates acetylation of Hsp90-K407/K419. In the absence of Tsc1, Hsp90 is hypoacetylated at these residues and binds less avidly to its inhibitors. HDAC inhibitor treatment restores Hsp90 acetylation and bladder cancer cell sensitivity to Hsp90 inhibitors.

Mutation and inactivation of *TSC1* in bladder cancer cells leads to decreased sensitivity to Hsp90 inhibitors. It appears that loss of Tsc1 may mediate this effect through Hsp90 hypoacetylation, which subsequently decreases Hsp90 inhibitor binding. We therefore treated the bladder cancer cells with a pan-HDAC inhibitor with the goal of restoring Hsp90 acetylation in Tsc1-mutant cells and subsequent sensitivity to Hsp90 inhibitors. Our initial data showed that Tsc1 mutated RT4 cells are more sensitive to the pan-HDAC inhibitor ACY-241 than WT Tsc1 T24 or UM-UC-3 cells (Figure 5A), presumably due to hyperactivity of HDACs. We next treated RT4 cells with 50nM ACY-241 for 16 hours, followed by 500 nM ganetespib for further 48 hours. We further show that the co-treatment of ACY-241 and ganetespib yields a combination index score <1, (0.79412; Supplementary Figure 5A). Our results demonstrated synergistic effect of combination HDAC and Hsp90 inhibitor treatment on RT4 cell proliferation (Figure 5B). Of note, the inhibitor doses used in the combination treatment were significantly lower than the IC50s of these drugs for RT4 cells. Additionally, combination treatment led to increased apoptosis as observed by immunoblot of cleaved caspase-3 (Figure 5C, Supplementary Figure 5B). We further confirmed that the sensitization of RT4 cells towards Hsp90 inhibitors as a result of HDAC co-inhibition was due to enhanced affinity of Hsp90 for ganetespib using biotinylated ganetespib binding assay (Figure 5D). Overall, our data provide evidence that inhibition of HDACs in bladder cancer cells that lack Tsc1 provides a strategy to enhance the efficacy of Hsp90 inhibitors.

## DISCUSSION

The tumor suppressor *TSC1* is a new co-chaperone of Hsp90 that modulates Hsp90 activity and enhances Hsp90 binding to its inhibitors [17, 20]. Mutations of *TSC1* have been identified in 14.5% of bladder tumors and loss of heterozygosity of *TSC1* has been reported approximately 54% of bladder cancers [21–26]. In this study we showed that mutation and loss of *TSC1* in bladder cancer cells reduced the accumulation of Hsp90 inhibitors in these cells and decreased cell sensitivity to Hsp90 inhibitors. We were able to rescue this effect by reintroducing WT Tsc1 suggesting that it is a Tsc1-specific effect.

Previous work by our group and others has shown that PTM of Hsp90 can modulate its binding and sensitivity to Hsp90 inhibitors [28–30, 33–35], we therefore asked whether Hsp90 was differentially modified in the absence of Tsc1. Mechanistically, we demonstrated that presence of Tsc1 resulted in acetylation of two lysine residues (K407/K419) near the catalytic site in the middle-domain of Hsp90. Mutation or absence of *TSC1* led to hypoacetylation of Hsp90 and this was demonstrated in Hsp90 isolated from four cell types lacking *TSC1*: 1) bladder cancer cells with *TSC1* mutation, 2) *TSC1* KO HAP1 cells, 3) conditional knockout of *TSC1* in mouse brain and 4) *TSC1* KO MEFs. Interestingly the effect of the Tsc1 mutation on Hsp90 acetylation appears to be Tsc1 specific and independent of the mTOR pathway, Hsp90 acetylation was unaffected both in *TSC2* KO MEFs and in HEK293 cells treated with the mTOR inhibitor rapamycin.

Hypoacetylation of Hsp90 enhances its ATPase activity and reduces binding to its inhibitors. Determination of precisely how Tsc1 loss leads to hypoacetylation of Hsp90 and which HAT or HDAC enzymes are involved will require further investigation. However, increasing Hsp90 acetylation through HDAC inhibition with ACY-241 resulted in enhanced Hsp90 inhibitor binding as well as sensitization to Hsp90 inhibitors in the *TSC1* mutated RT4 bladder cancer cell line.

HDAC6 mediated deacetylation of Hsp90 and regulation of its chaperone function has been reported previously [31, 32]. In fact our data are also in agreement with those published previously, confirming acetylation of Hsp90 leads to its inhibition. Therefore based on our findings presented here we would like to propose the following model, where in the presence of Tsc1 co-chaperone Hsp90 is acetylated and has a high affinity towards its N-domain inhibitors such as ganetespib (Figure 5E). Mutation and inactivation of *TSC1,* such as those frequently observed in bladder cancer cells leads to hypoacetylation of Hsp90, enhanced ATPase activity, and reduced binding to inhibitors (Figure 5E). This also reduces the sensitivity of bladder cancer cells to the Hsp90 inhibitors. This suggests that *TSC1* status may predict sensitivity to Hsp90 inhibitors in bladder cancer. Additionally, in those patients with *TSC1* mutated bladder cancer, inhibition of HDACs can potentially restore Hsp90 acetylation and sensitivity to Hsp90 inhibitors.

## MATERIALS AND METHODS

### Mice

All mouse experiments were performed under the ethical guidelines of the Washington University School of Medicine, and animal protocols were reviewed and approved by the Washington University School of Medicine Institutional Animal Care and Use Committee (IACUC #A-3381-01; Protocol #20160091). All mice used in this study were obtained from an existing breeding colony of *Tsc1*^GFAP^CKO mice (Uhlmann et al, 2002; Zeng et al, 2008) in the animal facility of the Washington University School of Medicine.

### Mammalian cell culture

Human embryonic kidney (HEK293), T24, UM-UC-3, and RT4 cells were acquired from the American Type Culture Collection (ATCC). *TSC1* KO, *HSP90α* KO, and wild-type HAP1 cells were acquired from Horizon Discovery. HEK293, UM-UC-3, WT MEF, *TSC1* KO and *TSC2* KO MEFs cells were grown in Dulbecco’s Modified Eagle Medium (DMEM, Sigma–Aldrich), T24 and RT4 cells were grown in McCoy’s 5A Medium (Sigma–Aldrich) and HAP1 cells were grown in Isocove’s Modified Dulbecco’s Medium (IMDM, Gibco) supplemented with 10% fetal bovine serum (FBS, Sigma–Aldrich). Cells were grown in a CellQ incubator (Panasonic Healthcare) at 37° C in an atmosphere containing 5% CO_2_.

### Plasmids

Tsc1-FLAG was cloned into pcDNA3 using Tsc1-FLAG-F–tatgcgggtaccatggattacaaggatgacgacgataagggagcccaacaagcaaatgtcggggagcttc; and Tsc1-FLAG-R–tatgcggggcccttagctgtgttcatgatgagtc. Tsc1-FLAG-L557Cfs, HSP90α-FLAG-K407A, HSP90α-FLAG-K419A, and HSP90α-FLAG-K407/K419A were generated by site-directed mutagenesis using the following primers: Tsc1-FLAG-L557Cfs-F–actcccatagactgccctgcggc; Tsc1-FLAG-L557Cfs-R–gccgcagggcagtctatgggagt; Hsp90α-FLAG-K407A-F–atgttgcaacaaagcgctattttgaaagttatc; Hsp90α-FLAG-K407A-R–gataactttcaaaatagcgctttgttgcaacat; Hsp90α-FLAG-K419A-F–aatttggtcaaagcttgcttagaactc; Hsp90α-FLAG-K419A-R–gagttctaagcaagctttgaccaaatt. Mutations were confirmed by DNA sequencing.

### Transient transfection and siRNA knock-down

HEK293 cells were transiently transfected with each construct using TransIT^®^-2020 (Mirus) transfection reagent according to company protocol and incubated at 37° C for 16 hours prior to protein extraction. Short interfering RNA (siRNA) scramble control and *TSC1* (Tsc1) targeting duplexes were purchased from GE Dharmacon and suspended in provided buffer. For Tsc1 knock-down, either 30 nM of control siRNA or 10 nM of each Tsc1 siRNA duplex (A, B and C) were mixed prior to transfection. Cells were incubated at 37° C then harvested for protein extraction (see below) or placed into downstream applications as described below.

### Protein extraction, immunoprecipitation, and immunoblotting

Protein extraction from mammalian cells was carried out using methods previously described [30]. For immunoprecipitation, protein lysates were incubated with anti-FLAG M2 Affinity Gel agarose (Sigma) for 2 hr at 4° C. Alternatively, for endogenous immunoprecipitation, protein lysate was incubated with Hsp90 antibody for 2 hr followed by incubation with protein G agarose (Qiagen) for 2 hr at 4° C. Immunopellets were washed 4 times with fresh lysis buffer (20mM Tris (pH7.4), 100 mM NaCl, 1 mM MgCl_2_, 0.1% NP40, protease inhibitor cocktail (Roche), and PhosSTOP (Roche)) and eluted with 5× Laemmli buffer. Precipitated proteins were separated by SDS-PAGE and transferred to nitrocellulose membranes. Co-immunoprecipitated proteins were detected by immunoblotting with indicated dilutions of antibodies. Primary antibodies recognizing FLAG 1:8000 (ThermoFisher Scientific; PA1-984B), Hsp90-835-16F1 1:8000 (ENZO Life Sciences; ADI-SPA-835), GAPDH 1:8,000 (ENZO Life Sciences; ADI-CSA-335), Tsc1 1:1000 (Cell Signaling Technology (CST); 4906), Tsc2 1:1000 (CST; 3990), K-Ac 1:1000 (CST; 9441), phos-Ser 1:1000 (Sigma; P5747), phos-Thr 1:1000 (Sigma; P6623), phos-Tyr 1:1000 (Millipore; 05-321), Phospho-p70 S6 Kinase (Thr389) (108D2) 1:2000 (CST; 9234), p70S6K 1:6000 (SantaCruz Biotechnology; sc-8418), and cleaved-caspase-3 1:1000 (CST; 9661) were used for immunoblotting. Endogenous Hsp90 was immunoprecipitated with Hsp90-835-16F1 (ENZO Life Sciences; ADI-SPA-835) at 1:1000 dilution. HRP-conjugated secondary antibodies raised against mouse, rabbit, and rat (Cell Signaling Technology) were used at 1:4000 dilution.

### Fluorescence microscopy

T24 and UM-UC-3 cells were transiently transfected with siRNA targeting Tsc1 and RT4 cells were transfected with Tsc1-FLAG for 48 hr as described above, then trypsinized and plated overnight on glass coverslips (#1). After 16 hr, BODIPY-ganetespib was added to cells at the indicated concentrations for an additional 4 hr. Cells were fixed in 4% paraformaldehyde for 20 minutes at room temperature and washed 3× with fresh PBS. Coverslips were then mounted onto glass slides using ProLong^®^ Gold antifade mounting media with DAPI (4′,6-diamidino-2-phenylindole) (ThermoFisher Scientific). Images were obtained using a Zeiss LSM780 confocal microscope.

### Cell proliferation colorimetric (WST) assay

T24, UM-UC-3 were seeded at 5,000 cell/well after siRNA *TSC1* knock-down and RT4 cells were seeded at 10,000 cells/well alone or after Tsc1-FLAG expression as described above in 96-well plates. Cells were treated with indicated concentrations of ACY-241 (Celgene Corporation) and/or ganetespib as indicated. After 72 hr, cell proliferation colorimetric (WST) assay was performed according to the manufacturer’s protocol (BioVision, Cat# K302-500). In brief, 10 μl WST was added to each well and the plate was returned to the 37° C incubator. After 60 minutes, absorbance at 450nm was measured on a Tecan Infinite M200 Pro and proliferation rate was calculated. For transfected/siRNA knockdown cells, transfections were incubated for 48 hours prior to trypsinization and plating in the 96-well plate.

### Hsp90 ATPase assay

ATPase activity of Hsp90 was measured as previously described (Kamal et al. 2003) with the following additional details. Following protein extraction and immunoprecipitation as described above in the Materials and Methods, protein-bound Protein-G agarose (Qiagen) was washed five times in 0.5M NaCl and 1% NP-40 buffer. Proteins were eluted from the beads twice with 50μl 0.1M glycine pH 3.0 and immediately neutralized using an equal volume of Tris pH 8.0. Protein was then concentrated with Amicon^®^ Ultra-2 mL, 10K centrifugal filters (Millipore). Using the Micro BCA^TM^ Protein Assay Kit (Thermo Scientific), protein was quantified to standardize the amount of protein used in the assay. Assay was performed as described in the P_i_Per^TM^ Phosphate Assay Kit instructions for use (Life Technologies). Standard curve with linear fit line was created from 0-100μM final concentration reactions. Hsp90 was incubated at 37°C for 1 hr with 100mM ATP as substrate, with or without 10μM ganetespib (Synta Pharmaceuticals). ATP turnover was calculated as mmol P_i_ per mol Hsp90 per minute, and relative ATPase activity was calculated from those values, with the value of Hsp90α alone representing 100% activity.

### Quantification and statistical analysis

The relationship of drug co-treatment was measured through an isobologram. The ED_50_ of each drug was plotted in Cartesian co-ordinates on an X, Y graph and a best fit line drawn. The co-ordinates of the ED_50_ for the co-treatment of the drugs was further plotted. The offset of the combined drugs from the best fit line is given as the combination index with equation: $CI=\frac{\left[A\right]}{ED50[A]}+\frac{\left[B\right]}{ED50[B]}$. The data presented are the representative or examples of three biological replicates unless it is specified. Data were analyzed with unpaired Student’s *t*-test. Asterisks in figures indicate significant differences (^*^ *p* < 0.05, ^**^ *p* < 0.01, ^***^ *p* < 0.001, and ^***^ *p* < 0.0001). Error bars represent the standard deviation (S. D.) for three independent experiments, unless it is indicated.

## SUPPLEMENTARY MATERIALS


