# Supplementary material for: Mutation of the co-chaperone Tsc1 in bladder cancer diminishes Hsp90 acetylation and reduces drug sensitivity and selectivity
Source: Oncotarget. 2019 Oct 8;10(56):5824–34. doi: 10.18632/oncotarget.27217 (PMC6791385; doi:10.18632/oncotarget.27217)
Supplement: Supplementary file 1 [file oncotarget-10-5824-s001.pdf]

Mutation of the co-chaperone Tsc1 in bladder cancer diminishes Hsp90 acetylation and reduces drug sensitivity and selectivity

SUPPLEMENTARY MATERIALS

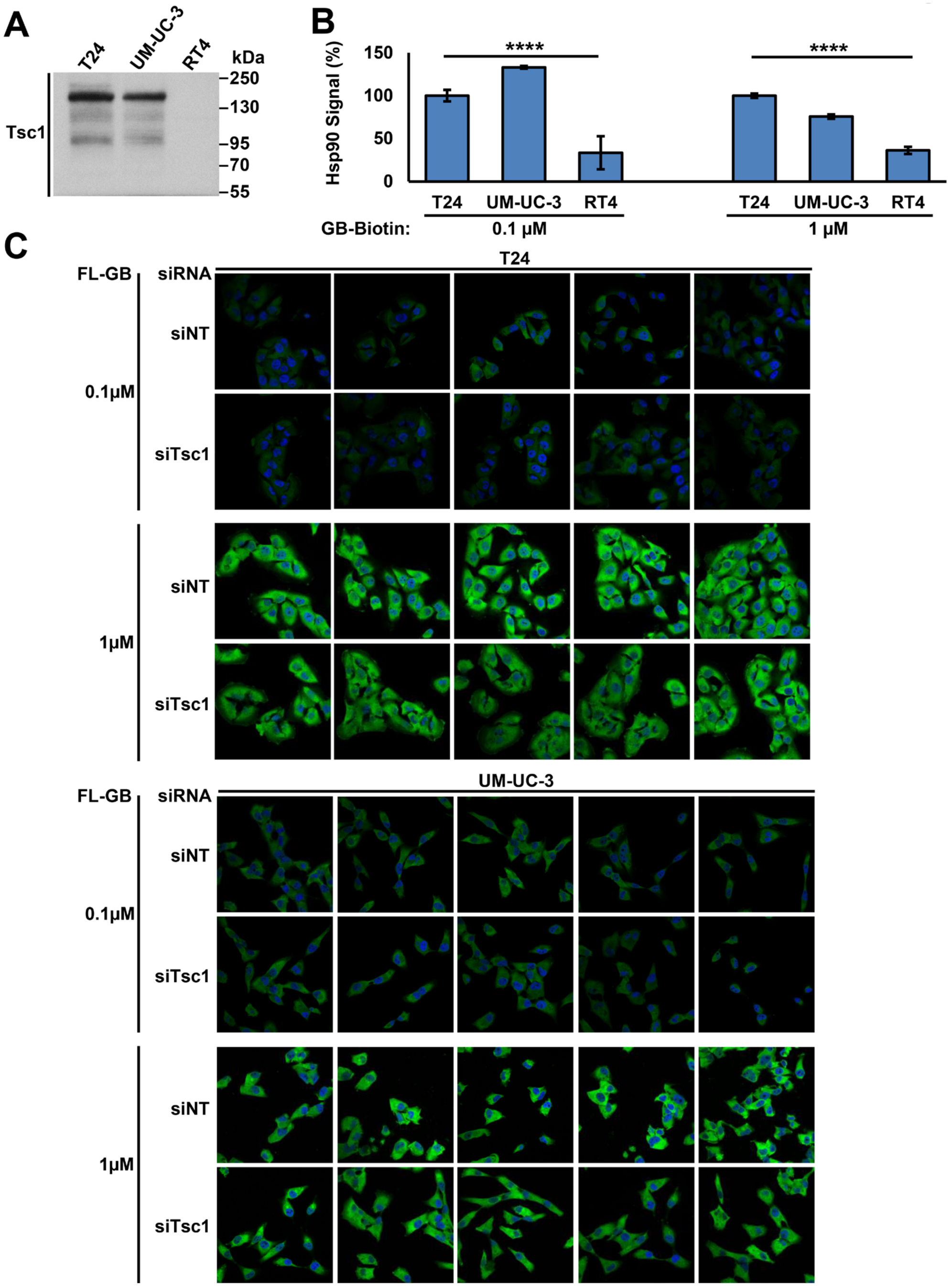

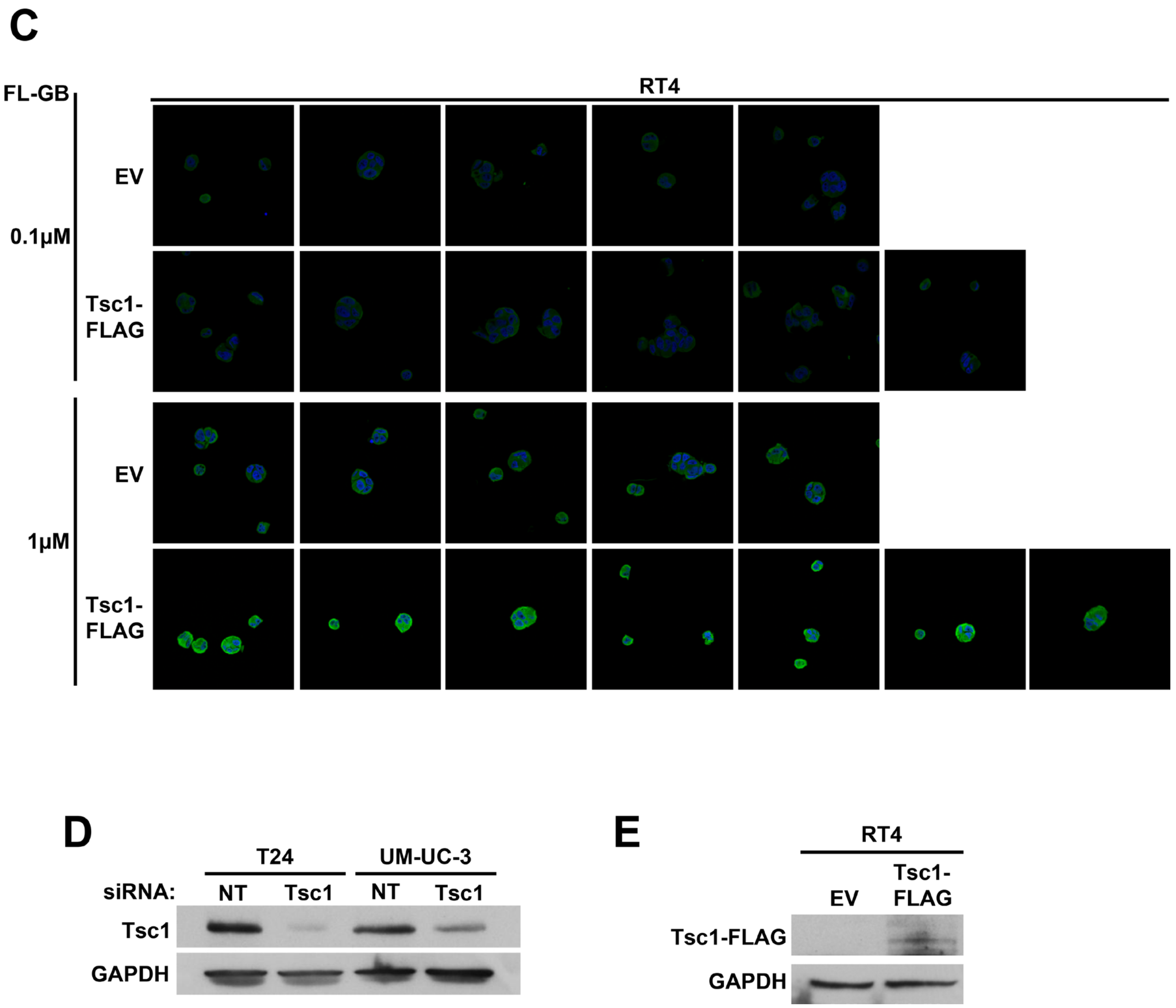

Supplementary Figure 1: Tsc1 expression determines Hsp90 inhibitor accumulation and sensitivity in bladder cancer cells. Related to Figure 1. (**A**) Endogenous Tsc1 levels in T24, UM-UC-3 and RT4 bladder cancer cell lines from Figure 1A. (**B**) Densitometry of the immunoblot for Hsp90 in Figure 1B. Error bars correspond to SD of three measurements. (**C**) Multiple confocal microscope images corresponding to Figure 1C that were used to quantify average fluorescence intensity in Figure 1D. (**D**) Immunoblot of siRNA knockdown of Tsc1 in T24 and UM-UC-3 used in Figure 1E. (**E**) Immunoblot of Tsc1-FLAG overexpression in RT4 used in Figure 1E.

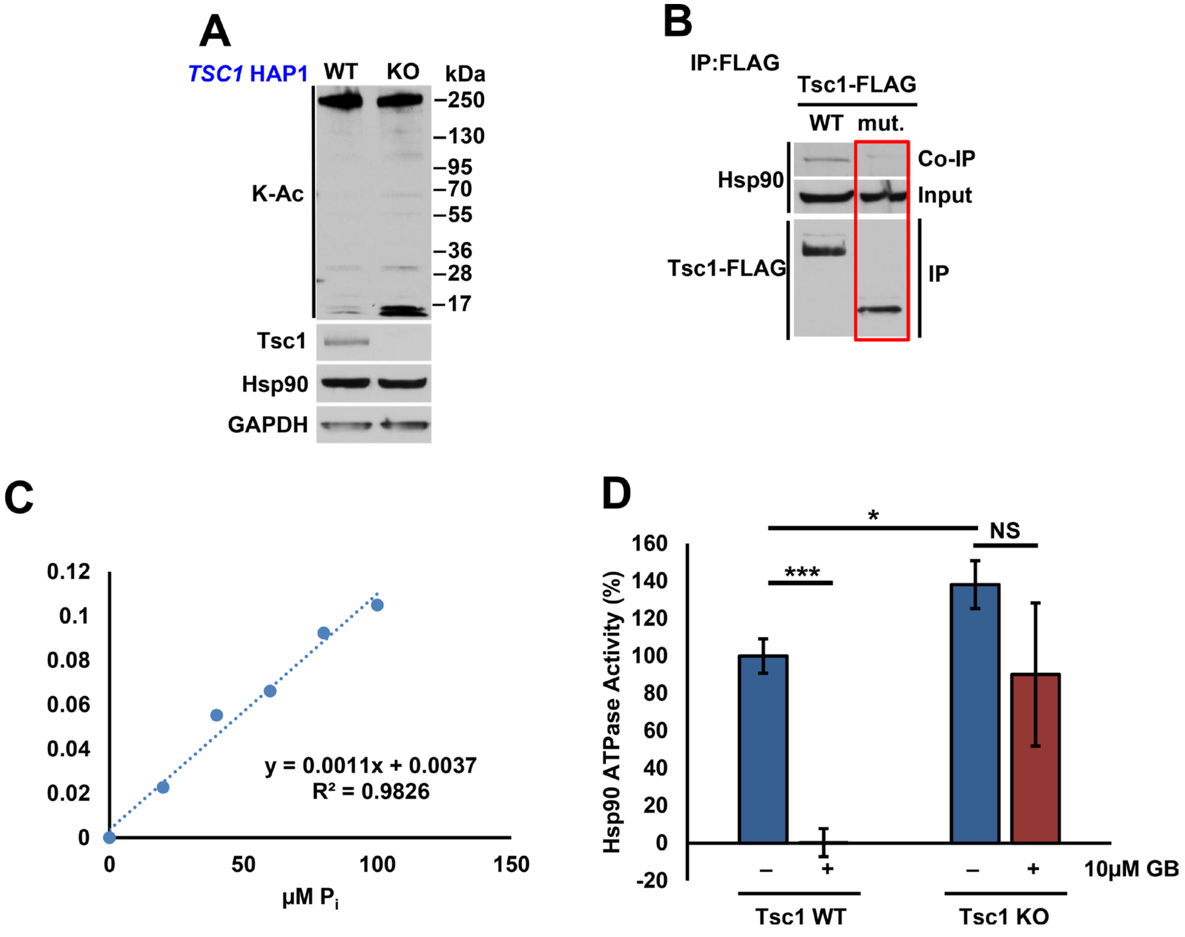

Supplementary Figure 2: Tsc1 facilitates acetylation of Hsp90. Related to Figure 2. (**A**) Lysine acetylation in whole cell lysate from TSC1 WT and TSC1 KO HAP1 cells. (**B**) Transient expression of Tsc1-WT-FLAG and Tsc1-L557Cfs-FLAG (mut.) in HEK293 cells followed by immunoprecipitation and immunoblot, corresponding to Figure 2B. (**C**) Standard curve of Pi used to determine Hsp90 ATPase activity. (**D**) Percent ATPase activity of Hsp90 isolated from TSC1 WT and TSC1 KO HAP1 cells. 10μM ganetespib is used as a control, corresponding to Figure 2E. A Student's *t*-test was performed to assess statistical significance (n.s., not significant; $*p < 0.05$; $^{***}p < 0.001$).

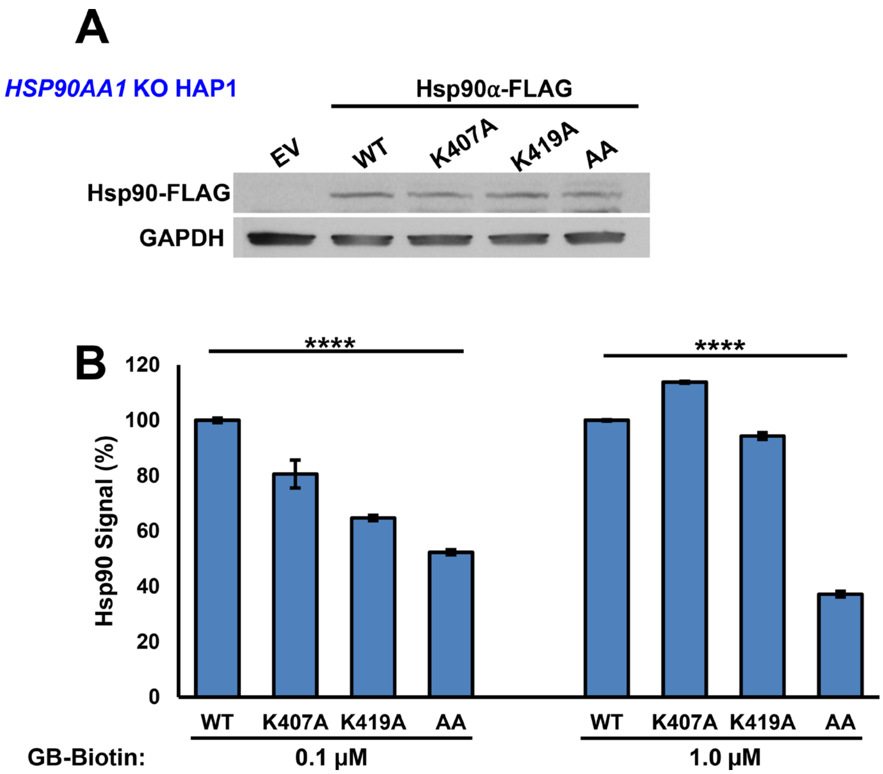

Supplementary Figure 3: Tsc1 facilitates acetylation of Hsp90-K407/K419. Related to Figure 3. (**A**) Immunoblot inputs of FLAG-tagged Hsp90α-WT, K407A, K419A, and K407/K419A transfected into HSP90α KO HAP1 cells, corresponding to Figure 3B. (**B**) Densitometry of the immunoblot for Hsp90 in Figure 3E. Error bars correspond to SD of three measurements.

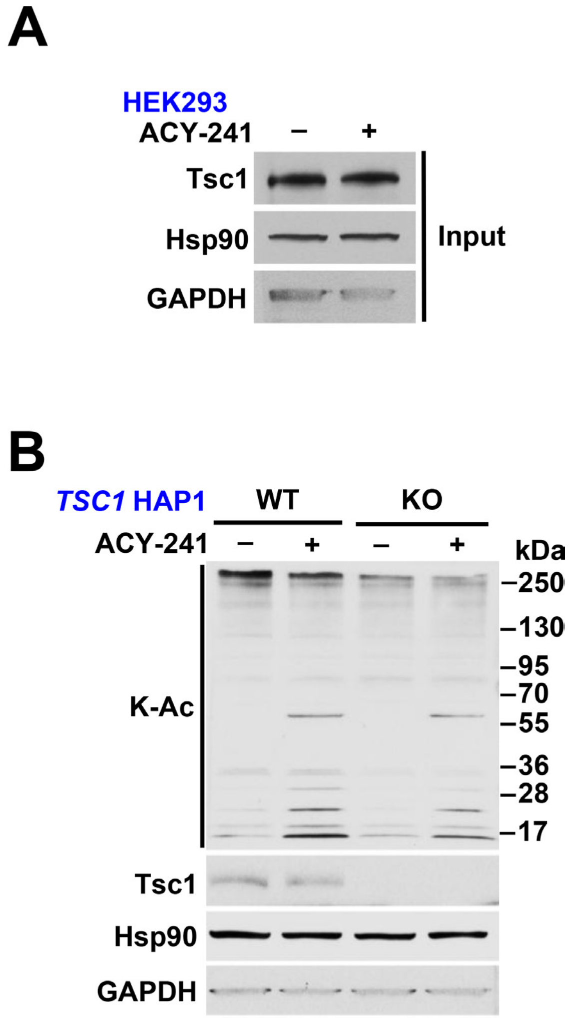

Supplementary Figure 4: HDAC inhibition rescues Hsp90 acetylation in TSC1-knock out cells. Related to Figure 4. (**A**) Endogenous Tsc1 and Hsp90 from HEK293 cells treated with or without 1 μM ACY-241 for 16 hr was examined by immunoblot; GAPDH was used as a loading control, corresponding to Figure 4A. (**B**) Lysine acetylation in whole cell lysate from TSC1 WT and TSC1 KO HAP1 cells treated with or without 1 μM ACY-241 for 16 hr was examined by immunoblot; GAPDH was used as a loading control, corresponding to Figure 4C.

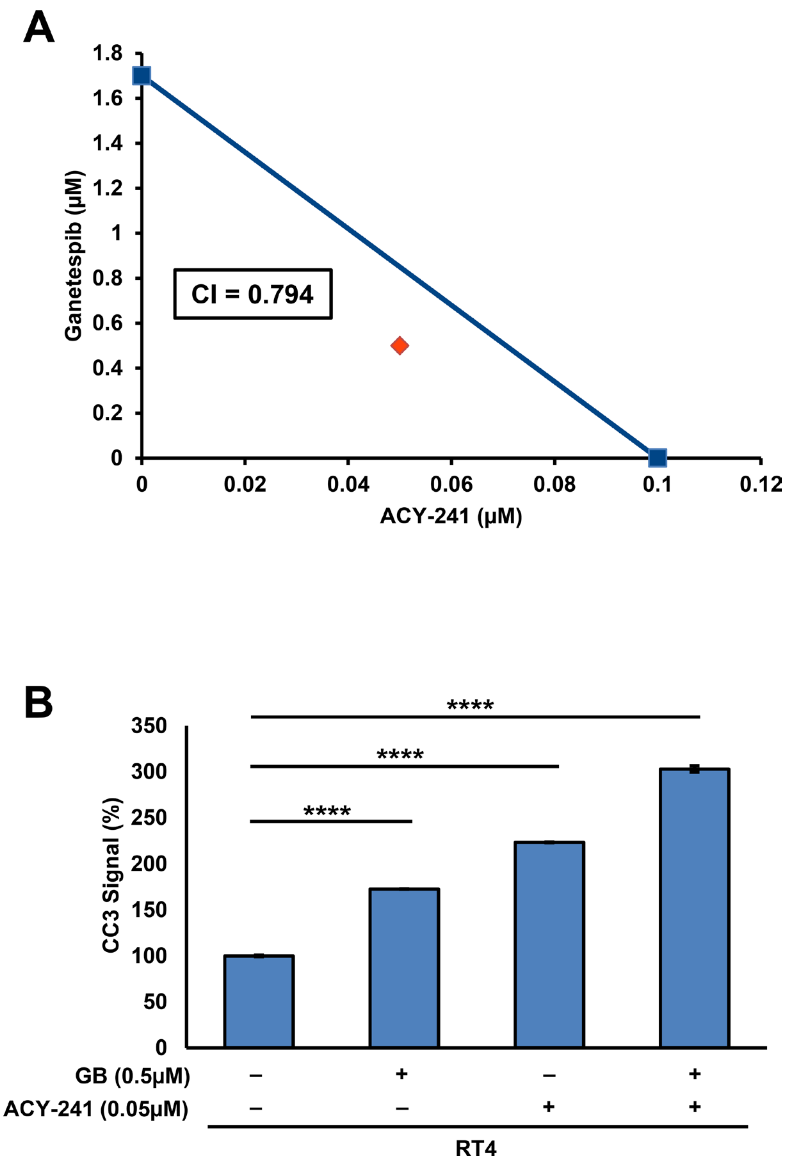

Supplementary Figure 5: HDAC inhibition synergizes with Hsp90 inhibition to induce apoptosis in bladder cancer. Related to Figure 5. (**A**) Isobologram of GB and ACY-241 co-treatment from Figure 5B. ED50 of GB, 1.7 µM, and ACY, 0.1 µM, were plotted as Cartesian co-ordinates with best fit line. The co-treatment ED50 of 0.5 µM (GB) and 0.05µM (ACY-241) was also plotted, and a combination index (CI) was calculated based on the offset of the co-treatment from best fit line. (**B**) Densitometry of the immunoblot for cleaved caspase-3 in Figure 5C. Error bars correspond to SD of three measurements. A Student's t-test was performed to asses statistical significance ($^{****}p$ > 0.0001).
